# Mental State Understanding and Moral Judgment in Children with Autistic Spectrum Disorder

**DOI:** 10.3389/fpsyg.2016.01478

**Published:** 2016-09-27

**Authors:** Francesco Margoni, Luca Surian

**Affiliations:** Department of Psychology and Cognitive Sciences, University of TrentoRovereto, Italy

**Keywords:** moral judgment, mental state understanding, theory of mind, autism spectrum disorders, moral development

Do children with autistic spectrum disorder (ASD) develop the ability to take into account an agent's mental states when they are judging the morality of his or her actions? The present article aims to answer this question by reviewing recent evidence on moral reasoning on children with autism and typical development. A basic moral judgment (e.g., judgments of violations in which negative intentions are followed by negative consequences) and the ability to distinguish between conventional and moral violations appear to be spared in autism (Leslie et al., 2006). However, a closer look at the data reveals that these capacities can be explained by the tendency of ASD individuals to rely heavily on actions consequences and other external factors rather than agents' mental states. By contrast, studies that presented typically developing (TD) children with accidental and failed attempts actions have shown that even preschoolers can display an intent-based moral judgment (e.g., Cushman et al., 2013; Margoni and Surian, 2016). The tendency to rely on outcome in ASD children is further confirmed by those studies that direcly show that ASD individuals fail to attend to the agents' intentions when the cases are more complex or ambiguous, like in accidentally harmful actions or failed attempts to harm. We propose that the impairment in understanding others' mind hinders the development of an intent-based moral judgment in children with ASD.

## Mental state reasoning in the moral judgment tasks

In our social life, we often engange in the evaluation of others' actions and intentions, and we are very sensitive to harmful acts and violations of rights. For example, we maintain friendships on the basis on an assessment of our friends' moral behaviors toward us. The production and the justification of a moral judgment is a complex socio-cognitive task that often requires the use of mental state reasoning abilities (Young et al., 2007; Moran et al., 2011). In particular when people are asked to evaluate accidental harming (or helping) actions or failed attempts to harm (or help), they need to weigh the agents' intention, that requires a mental state analysis, against the external consequences of the action. Several neuroscientific studies confirm the association between moral judgment and theory of mind (Young et al., 2007, 2010; Young and Saxe, 2009).

Then, to what extent individuals with ASD, who present deficits in theory of mind abilities (Baron-Cohen et al., 1985, 2000; Bowler, 1992; Surian and Leslie, 1999; Abell et al., 2000; Castelli et al., 2002), meet with difficulties in the acquisition of an intent-based moral judgment? Individuals with ASD are characterized by impaired social interactions and communication abilities, and a set of restricted and repetitive behaviors. Here we focus on their impairment in mentalizing, that has been shown to be a main factor affecting their socio-moral abilities. Studies on the moral judgment of ASD children have traditionally focused on (a) the capacity to distinguish between moral and social-conventional transgressions and (b) the ways in which individuals with autism judge the moral rightness or wrongness of an action.

## Moral and conventional transgressions

One fundamental aspect of the moral competence has been identified by social domain theorists in the capacity to distinguish between moral and social-conventional violations. While the former involve a victim and are to be blamed regardless of the social context, the latter do not need to involve a victim and are contingent over a specific group consensus or authority mandate (Turiel, 1978; Nucci, 1981; Killen and Smetana, 2015). By the age of three, children judge moral violations, like hit someone, more harshly, and less authority-dependent than social-conventional, like wearing pajamas at school (Nucci, 1985; Smetana and Braeges, 1990).

The capacity to distinguish between these two types of violation is intact in ASD individuals (Blair, 1996; Rogers et al., 2006; Zalla et al., 2011; Shulman et al., 2012). However, ASD individuals produce poorer justifications compared to TD individuals, and they do not evaluate moral violations as more serious than non-moral but disgusting actions, such as drinking tomato soup out of the bowl at a dinner party. Moreover, contrary to TD children, school-aged children with ASD are swayed by the victims' emotion and judge wrong actions that caused the crying of the victim more harshly than wrong actions that did not cause any crying (Weisberg and Leslie, 2012). ASD children usually succeed in tasks devised to investigate the moral-conventional distinction, but they rely mainly on external factors that could depend on irrelevant variables such as the particular emotional level of the agents.

## The relative weight of intention and outcome in the judgments of ASD individuals

A working hypothesis here is that ASD children respond as TD children do when they are presented with simple, unambiguous moral cases (i.e., a negative/positive outcome produced by an intentional action with the same valence). In those cases, the difficulties encountered in integrating the mental state understanding in the moral reasoning can be overcome by the children's reliance on action outcomes and victims' emotional reactions. For this reason, ASD children appear to develop a basic moral judgment.

ASD school-aged children evaluate actions that are motivated by positive or negative intentions and are followed by congruent outcomes as TD children do (Leslie et al., 2006; Li et al., 2014). Moreover, they are able to judge an agent that caused intentionally a bad outcome more harshly than an agent that caused it accidentally, although they do not produce verbal justifications that refer to the agent's intention (Grant et al., 2005). However, Steele et al. (2003) found that children with ASD aged 4–14 failed to distinguish between intentional and accidental bad acts (e.g., failing to come to a planned meeting as a result of canceling the plan without telling or as a result of the bus breaking). Studies on ASD adults also showed that they judge an accidental harm both more punishable and more intentional compared to TD adults, suggesting a partial impairment in the ability to rely on intentions (Buon et al., 2013; see also Rogé and Mullet, 2011; Zalla and Leboyer, 2011; Salvano-Pardieu et al., 2015). Nevertheless, ASD school-aged children distinguish between a distressed victim and an individual in distress that however is not a victim (Leslie et al., 2006). So, their judgments do not completely rely on the external outcomes assessment.

However, what about the judgments of more complex cases such as the failed attempts to help or harm, that require a more substantial contribution of mental state reasoning? In fact, in judging an ambiguous case such as a failed attempt to harm, it is not possible to rely solely on action outcomes, and still produce a moral condemnation of the agent.

**Figure 1:**
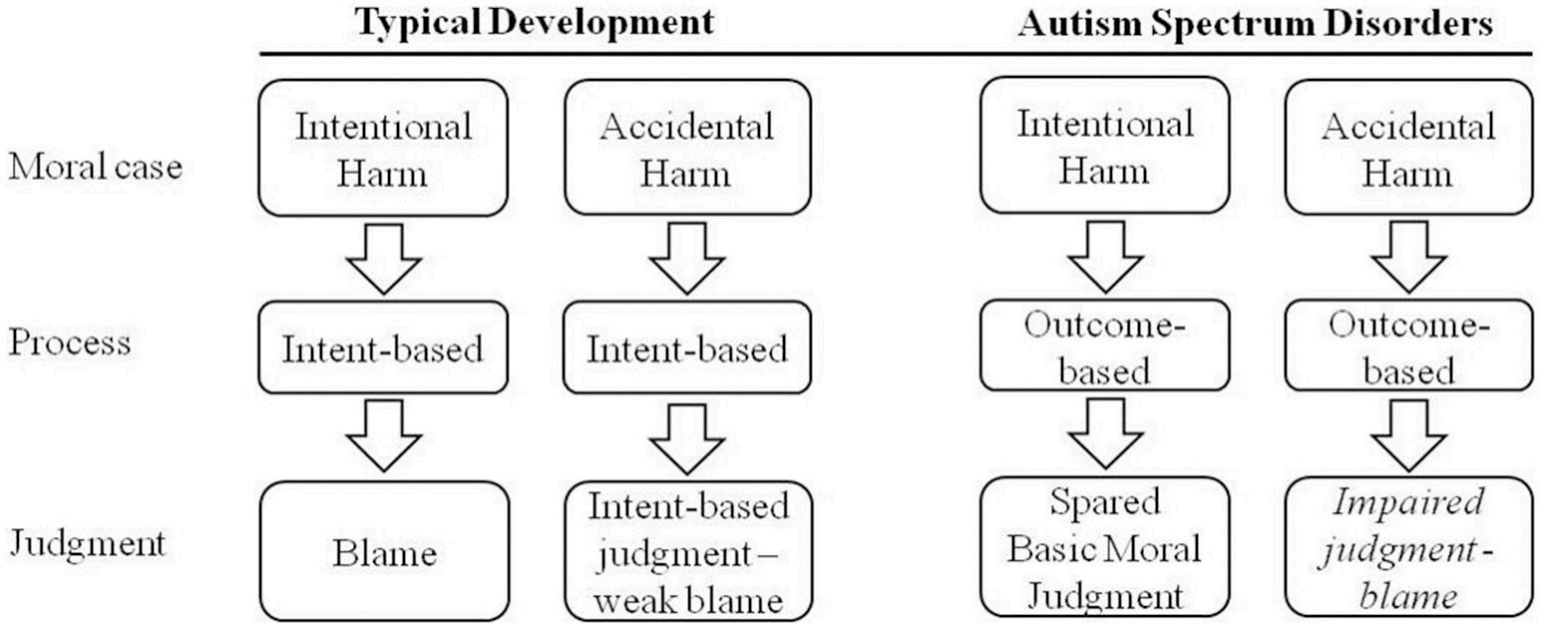
**Main results concerning the mental state reasoning in ASD individuals' moral reasoning**.

A first evidence of an outcome-bias in the judgment in ASD individuals comes from those studies that reported a “heteronomous” (i.e., rules are understood as handed down by authority, and violations are wrong because they produce bad outcomes, namely they lead to punishment) rather than an “autonomous” (i.e., rules are based on socially agreed-on principles, and violations are wrong because of the agent's beliefs and motivations) moral reasoning in ASD school-aged children (Grant et al., 2005; Takeda et al., 2007; see also Fadda et al., 2016). ASD children attributed moral wrongness and badness to actions that caused bad outcomes. A second and more direct evidence comes from a study that presented ASD individuals with accidental and failed attempted harms. Moran et al. (2011) found that they failed to distinguish between the two scenarios, and they judged the accidental harm significantly more harshly than TD individuals. Moreover, there is evidence of an activation of the right temporo-parietal junction (RTPJ)—an area associated with mental state reasoning—in TD individuals during the evaluation of intentional vs. accidental harm, but such result has not been found in adults with ASD (Koster-Hale et al., 2013). These results clearly suggest that ASD individuals fail to integrate the agent's mental states in their moral reasoning when judging situations in which intentions and outcomes present different valences (see Figure 1).

## Theoretical implications of the studies on mental reasoning in ASD individuals' moral judgments

Three main theoretical implications relevant for the current understanding of the relationship between theory of mind and moral reasoning could be inferred from the results we briefly discussed. First, the evidence that ASD individuals, who are characterized by an impaired mental state understanding, show an atypical moral judgment, further confirms that theory of mind is fundamental for the development of a mature moral reasoning.

Second, the study of moral judgment in ASD individuals could prove useful in assessing the role of cognitive empathy in the production of a moral evaluation. ASD individuals show a spared capacity for emotional empathy (e.g., Blair, 1999; Rogers et al., 2007), that is, the proper emotional response to others' emotions, but an impaired capacity for cognitive empathy, that is, the proper knowing how others may feel. While emotional empathy skills help ASD children developing a basic moral judgment by relying on the emotional and external aspects of the moral case such as the victims' emotional reactions or the actions outcomes (Leslie et al., 2006; Hobson et al., 2009; Weisberg and Leslie, 2012), the poor understanding of the cognitive aspects hinders the development of an intent-based moral judgment. Further studies confirm this interpretation by reporting that aspects related to cognitive empathy impairment affect the moral evaluations of ASD individuals (Channon et al., 2010; Gleichgerrcht et al., 2013; Patil et al., 2016).

A third relevant theoretical implication concerns whether the action understanding required in moral evaluation is mentalistic. A mentalistic understanding represents and explains others' actions by ascribing mental states such as beliefs, desires, and internal representations to the agents (Baron-Cohen et al., 1985; Leslie, 1987; Surian et al., 2007; Baillargeon et al., 2010). By contrast, a non-mentalistic or teleological understanding represents others' actions without ascribing mental states, by linking directly the agent's actions, the goal-states and the situational constraints through the principle of rational actions (i.e., agents act to achieve certain goals choosing the most efficient means; Gergely and Csibra, 2003; Schlottmann et al., 2009). According to the proponents of teleological accounts of action understanding, humans first develop very early in life a non-mentalistic understanding, and only later they acquire a mentalistic understanding. While it could be argued that ASD individuals possess the ability to interpret actions in a non-mentalistic way already during preschool years (Hamilton, 2009; Vivanti et al., 2011), we have seen that they do not develop a mature intent-based moral judgment. Therefore, the literature on ASD individuals suggests that a non-mentalistic understanding is not sufficient for the development of a full-blown intent-based moral reasoning.

## Conclusions

The ability to produce moral evaluations often requires the understanding of others' mental states and it is central for living in human social groups. While much more research is needed to acquire a full understanding of the development of moral judgment in ASD individuals, the current state of the literature suggests that this clinical population encounters some difficulties in developing a mature intent-based moral judgment because of the well-known impairment in mental state understanding. Nevertheless, ASD individuals show the ability to produce a basic moral judgment by relying on external cues such as the action outcomes and the victims' emotional reactions.

Can these results turn out to be useful in guiding programs designed to improve moral judgment in children with ASD? Since a main result of the literature we reviewed is that individuals with ASD show difficulties in integrating mental states information in their judgments, clinical treatments, and educational programs aimed at improving their theory of mind abilities are likely to have, as a side-effect, a positive impact also on their moral reasoning abilities. Further research is needed to point out whether such a desiderable effect is achieved equally by any effective training on mentalizing skills (e.g., Silver and Oakes, 2001; Fisher and Happé, 2005; Begeer et al., 2011), or it is best achieved by a program that requires both mental state attribution and the generation of moral judgments.

## Author contributions

All authors listed, have made substantial, direct and intellectual contribution to the work, and approved it for publication.

### Conflict of interest statement

The authors declare that the research was conducted in the absence of any commercial or financial relationships that could be construed as a potential conflict of interest.
