# Corrigendum: Mental State Understanding and Moral Judgment in Children with Autistic Spectrum Disorder

**DOI:** 10.3389/fpsyg.2016.01705

**Published:** 2016-10-27

**Authors:** Francesco Margoni, Luca Surian

**Affiliations:** Department of Psychology and Cognitive Sciences, University of TrentoRovereto, Italy

**Keywords:** moral judgment, mental state understanding, theory of mind, autism spectrum disorders, moral development

We realized that Figure 1 was misleading. The figure showed the mechanisms underlying the moral judgment of attempted harm cases in individuals with autistic spectrum disorder (ASD). However, it would be more in line with the current studies, that primarily presented ASD individuals with cases of accidental harm, to show in the figure the ASD individuals' processing of accidental harm. Therefore, we replaced “Attempted Harm” with “Accidental Harm” in the top boxes, and we also changed accordingly the bottom boxes. The authors apologize for the mistake.

**Figure 1:**
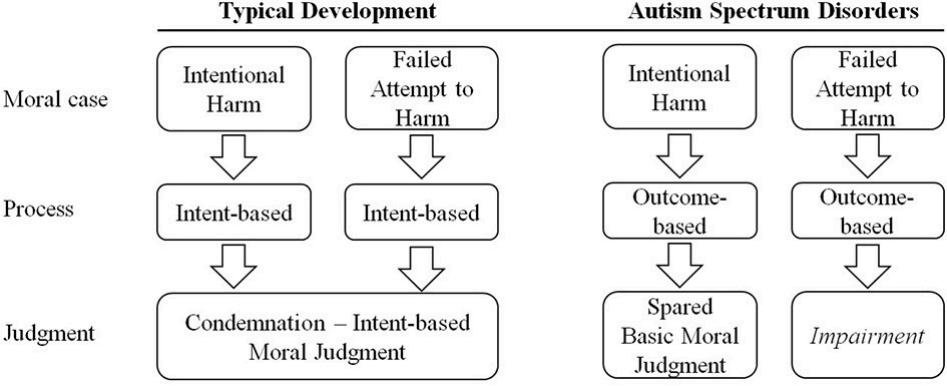
**A comparison of moral judgment in children with typical development and children with autism**.

The original article has been reproduced with the correct image, originally it was published with the version of Figure 1 displayed here.

The original files have been updated.

## Author contributions

All authors listed, have made substantial, direct and intellectual contribution to the work, and approved it for publication.

### Conflict of interest statement

The authors declare that the research was conducted in the absence of any commercial or financial relationships that could be construed as a potential conflict of interest.

